# Statistically Controlling for Confounding Constructs Is Harder than You Think

**DOI:** 10.1371/journal.pone.0152719

**Published:** 2016-03-31

**Authors:** Jacob Westfall, Tal Yarkoni

**Affiliations:** University of Texas at Austin, Austin, TX, United States of America; University of Vienna, School of Psychology, AUSTRIA

## Abstract

Social scientists often seek to demonstrate that a construct has *incremental validity* over and above other related constructs. However, these claims are typically supported by measurement-level models that fail to consider the effects of measurement (un)reliability. We use intuitive examples, Monte Carlo simulations, and a novel analytical framework to demonstrate that common strategies for establishing incremental construct validity using multiple regression analysis exhibit extremely high Type I error rates under parameter regimes common in many psychological domains. Counterintuitively, we find that error rates are highest—in some cases approaching 100%—when sample sizes are large and reliability is moderate. Our findings suggest that a potentially large proportion of incremental validity claims made in the literature are spurious. We present a web application (http://jakewestfall.org/ivy/) that readers can use to explore the statistical properties of these and other incremental validity arguments. We conclude by reviewing SEM-based statistical approaches that appropriately control the Type I error rate when attempting to establish incremental validity.

## Introduction

A common goal of statistical analysis in the social sciences is to draw inferences about the relative contributions of different variables to some outcome variable. When regressing academic performance, political affiliation, or vocabulary growth on other variables, researchers often wish to determine which variables matter to the prediction and which do not—typically by considering whether each variable’s contribution remains statistically significant after statistically controlling for other predictors. When a predictor variable in a multiple regression has a coefficient that differs significantly from zero, researchers typically conclude that the variable makes a “unique” contribution to the outcome. And because measured variables are typically viewed as proxies for latent constructs of substantive interest—for example, two cognitive ability measures might be taken to index spatial versus verbal ability—it is natural to generalize the operational conclusion to the latent variable level; that is, to conclude that the latent construct measured by a given predictor variable itself has *incremental validity* in predicting the outcome, over and above other latent constructs that were examined [1,2].

Incremental validity claims pervade the social and biomedical sciences. In some fields, these claims are often explicit. To take the present authors’ own field of psychology as an example, a Google Scholar search for the terms “incremental validity” AND psychology returns (in January 2016) over 18,000 hits—nearly 500 of which contained the phrase “incremental validity” in the title alone. More commonly, however, incremental validity claims are implicit—as when researchers claim that they have statistically “controlled” or “adjusted” for putative confounds—a practice that is exceedingly common in fields ranging from epidemiology to econometrics to behavioral neuroscience (a Google Scholar search for “after controlling for” and “after adjusting for” produces over 300,000 hits in each case). The sheer ubiquity of such appeals might well give one the impression that such claims are unobjectionable, and if anything, represent a foundational tool for drawing meaningful scientific inferences.

Unfortunately, incremental validity claims can be deeply problematic. As we demonstrate below, even small amounts of error in measured predictor variables can result in extremely poorly calibrated Type 1 error probabilities. This basic problem has been discussed in a number of literatures—most extensively, in epidemiology and biostatistics, where concerns about incremental validity claims are often discussed under the heading of *residual confounding* [3–5], but also in fields ranging from psychology to education to econometrics [6–11]. The common thread is that measurement unreliability and model misspecification will often have a deleterious and large effect on parameter estimates (and associated error rates) when covariates are entered into regression-based model. Consequently, under realistic assumptions, it can be shown that a large proportion of incremental validity claims in many disciplines are likely to be false.

In this paper, we develop and apply a general statistical and conceptual framework for understanding and evaluating claims about incremental validity. We begin by providing an intuitive statement of the problem using simple examples and simulated data. We discuss the most common forms of incremental validity argument and identify the unstated assumptions they rest on. Next, we introduce a formal statistical framework for analytically determining the expected Type I error rate of incremental validity claims as a function of key parameters like sample size, effect size, and reliability. We demonstrate that the likelihood of spurious inference is surprisingly high under real-world conditions, and often varies in counterintuitive ways across the parameter space. For example, we show that, because measurement error interacts in an insidious way with sample size, the probability of incorrectly rejecting the null and concluding that a particular construct contributes incrementally to an outcome quickly approaches 100% as the size of a study grows.

In the latter part of the paper, we consider potential solutions to the problems we have identified. We focus attention on structural equation modeling (SEM) methods that can maintain appropriate Type I error rates provided certain assumptions are met—or, alternatively, that can be used to identify the boundary conditions under which an observed association can be said to hold. We also provide a novel perspective on power analysis that takes the measurement unreliability of covariates into account, providing more realistic—and surprisingly large—estimates of the sample sizes typically required to support incremental validity claims. Taken as a whole, our work provides a formal framework for understanding the effects of multiple predictors on significance testing in the presence of unreliability, and offers practical guidelines for dealing with a very common, but largely unappreciated, problem.

## An Intuitive Statement of the Problem

Incremental validity claims come in a number of different forms. The most basic and common of these is what might be called the *argument for predictive utility*. Stated abstractly, it says: “If measurements of construct *X* correlate significantly with outcome *Y* even when controlling for existing measure(s) *Z*, then *X* is a useful predictor of *Y*, over and above *Z*.” As noted above, examples of this argument abound throughout the social and biomedical sciences. For example, epidemiologists have concluded that eating processed meat significantly increases colorectal cancer risk, on the basis of prospective studies that consistently find a positive association between the two variables when controlling for a host of confounding variables [12,13]. Organizational psychologists advocate the use of measures such as Emotional Intelligence on the grounds that they incrementally predict job performance when controlling for standard personality and cognitive ability measures [14,15]. Political scientists frequently seek to quantify the incremental contributions of specific demographic variables to voting preferences (e.g., are higher-income individuals more likely to vote Republican in US elections after controlling for differences in education level, race, state, etc.; [16–18]). And cognitive neuroscientists' arguments for the utility of brain-based predictive models are often predicated on those models’ putative ability to predict real-world outcomes (e.g., product purchases or smoking cessation) above and beyond relevant self-report variables [19,20].

In all of these cases—and thousands of others—the claims in question may seem unobjectionable at face value. After all, in any given analysis, there is a simple fact of the matter as to whether or not the unique contribution of one or more variables in a regression is statistically significant when controlling for other variables; what room is there for inferential error? Trouble arises, however, when researchers behave as if statistical conclusions obtained at the level of observed measures can be automatically generalized to the level of latent constructs [9,21]—a near-ubiquitous move, given that most scientists are not interested in prediction purely for prediction’s sake, and typically choose their measures precisely so as to stand in for latent constructs of interest. That is, researchers typically do not care to show that, say, school vouchers are associated with improved academic performance after controlling for a specific survey item asking about respondents’ income bracket; rather, the goal is to show that the vouchers may improve performance after accounting for the general construct of income (or, more generally, socioeconomic status).

To see the problem intuitively, consider a slight alteration of a familiar example from many introductory data analysis courses. Suppose we are given city statistics covering a four-month summer period, and observe that swimming pool deaths tend to increase on days when more ice cream is sold. As astute analysts, we immediately identify average daily temperature as a confound: on hotter days, people are more likely to both buy ice cream and visit swimming pools. Using multiple regression, we can statistically control for this confound, thereby eliminating the direct relationship between ice cream sales and swimming pool deaths.

**Fig 1:**
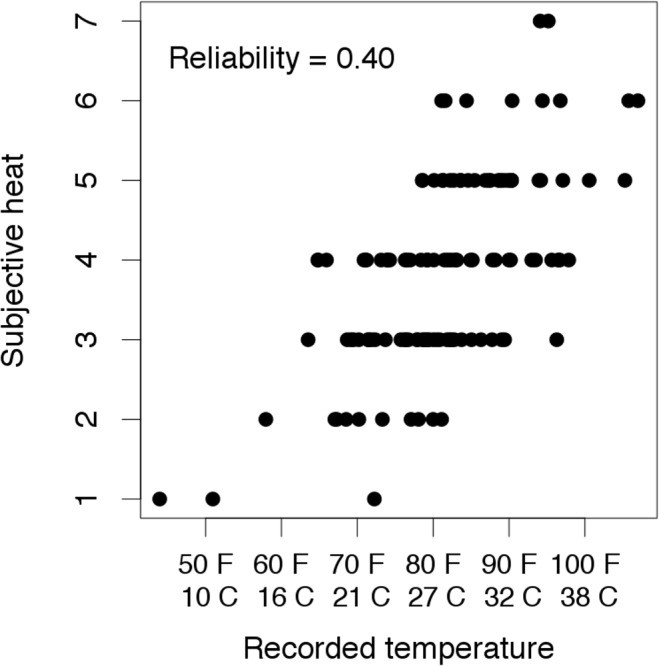
Plot of subjective heat ratings on a 7-point Likert scale against the “true” underlying daily temperatures.

**Fig 2:**
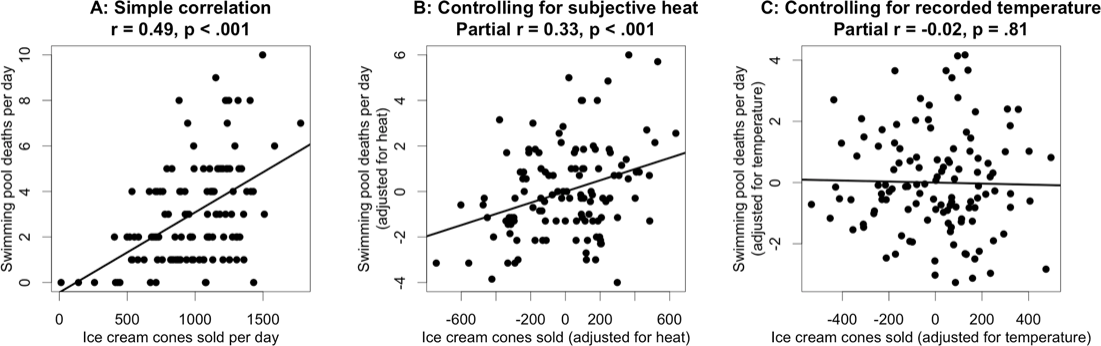
Illustration of residual confounding. (A) Simple relationship between daily swimming pool deaths and number of ice cream cones sold. (B) Relationship between daily swimming pool deaths and number of ice cream cones sold after controlling for subjective heat Likert ratings. (C) Relationship between daily swimming pool deaths and number of ice cream cones sold after controlling for recorded daily temperatures.

Now consider the following twist. Rather than directly observing recorded daily temperatures, suppose we obtain self-reported Likert ratings of subjectively perceived heat levels. A simulated batch of 120 such observations is illustrated in Fig 1, with the reliability of the subjective heat ratings set to 0.40—a fairly typical level of reliability for a single item in psychology. (A conventionally acceptable level of reliability for sum-scores derived from a measurement scale in psychology is around 0.8. If such a scale consists of six parallel items, which would be a fairly typical number of items in many contexts, then by the Spearman-Brown formula, the reliability of each individual item would be around 0.4.) Fig 2 illustrates what happens when the error-laden subjective heat ratings are used in place of the more precisely recorded daily temperatures. The simple relationship between ice cream sales and swimming pool deaths (Fig 2A) is positive and substantial, *r*(118) = .49, *p* < .001. When controlling for the subjective heat ratings (Fig 2B), the partial correlation between ice cream sales and swimming pool deaths is smaller, but remains positive and statistically significant, *r*(118) = .33, *p* < .001. Is the conclusion warranted that ice cream sales are a useful predictor of swimming pool deaths, over and above daily temperature? Obviously not. The problem is that subjective heat ratings are a noisy proxy for physical temperature, so controlling for the former does not equate observations on the latter. If we explicitly control for recorded daily temperatures (Fig 2C), the spurious relationship is eliminated, as we would intuitively expect, *r*(118) = -.02, *p* = .81.

The foregoing example is based on a single batch of simulated data. If we repeat the simulation 10,000 times with the same parameter values, we find a spurious partial correlation between ice cream sales and swimming pool deaths when controlling for subjective heat ratings 92% of the time.

While the variables in the above example were deliberately chosen so that the absurdity of the hypothetical relationship is clear, the parameter values upon which it is based, and the structure of the statistical argument itself, are representative of many common research situations. Table 1 presents expected Type I error rates for a number of other parameter regimes common to different scientific disciplines—ranging from small-sample lab-based experiments involving large effects (e.g., n = 30, r = 0.6) to population-level models involving tens of thousands of individuals and putatively small associations (n = 30,000, r = 0.2). In each case, we quantify the probability of (incorrectly) rejecting the null—that is, of concluding that a construct of interest makes a statistically significant contribution after controlling for a putative confound, when in fact the confound fully accounts for the relationship at the latent-variable level. For simplicity, we assume that measurement reliability is identical for both predictors.

**Table 1:** Type 1 error rates for a few parameter combinations. N = sample size; ES (r) = correlations of predictor with covariate and covariate with outcome; reliability = reliability of predictor and covariate. These error rates are determined using the methods described in the next section, and described in more detail in the S1 Appendix.

N	ES (r)	reliability	Type I error rate
30	0.6	0.4	0.12
30	0.6	0.8	0.07
120	0.5	0.4	0.2
120	0.5	0.8	0.09
300	0.4	0.4	0.19
300	0.4	0.8	0.09
3000	0.3	0.4	0.48
3000	0.3	0.8	0.16
30000	0.2	0.4	0.76
30000	0.2	0.8	0.25

While the Type I error rate varies considerably depending on sample size, effect size, and reliability, it is apparent from Table 1 that it is very often much larger than the nominal value of 5%. We submit that if there is a high probability of rejecting the null hypothesis in such situations even when it is actually true, then rejecting the null hypothesis cannot be considered convincing empirical evidence that a construct has incremental predictive utility. To be confident that an incremental validity argument is sound, one would need to either ensure perfect measurement reliability, or formally account for the potential effects of unreliability in one’s model. The former is a daunting—and usually impossible—proposition. The latter is quite feasible, but, as we discuss in a later section, cannot be accomplished with standard multiple regression.

## A General Statistical Framework for Assessing Incremental Validity

Having provided illustrative examples to prime readers’ intuitions, we now undertake a more comprehensive evaluation of the Type 1 error rates associated with incremental validity arguments. We first lay out the statistical models involved and define the relevant null hypotheses. We then quantify how the Type 1 error rates for tests of incremental validity claims vary across a broad range of parameter values. We have also written an interactive web application (“Ivy,” accessible online at http://jakewestfall.org/ivy/) that readers can use to explore the statistical properties of these and other incremental validity arguments for themselves. In S1 Appendix, we give the analytical derivations underlying these results, in which we determine the probabilities of rejecting different combinations of regression coefficients as a function of the simple or partial correlations among the outcome and the latent predictors, the reliabilities, and the sample size.

Consider a regression of an outcome *Y* on two true scores *T*_*j*_,

$$Y=\beta_{T0}+\beta_{T1}T_{1}+\beta_{T2}T_{2}+\;e_{T},$$

with *e*_*T*_ a random disturbance term (subscripts indexing people are omitted for simplicity). But rather than observing the latent predictors *T*_*j*_ directly, we instead observe two imperfectly measured indicators *X*_*j*_ = *b*_*j*_*T*_*j*_ + *e*_*j*_, so that the regression we actually observe is

$$Y=\beta_{X0}+\beta_{X1}X_{1}+\beta_{X2}X_{2}+\;e_{X}.$$

From these regressions we define the following parameters:

*ρ*_1_: The simple correlation between *Y* and *T*_1_.

*ρ*_1.2_: The partial correlation between *Y* and *T*_1_, controlling for *T*_2_.

*ρ*_2_: The simple correlation between *Y* and *T*_2_.

*ρ*_2.1_: The partial correlation between *Y* and *T*_2_, controlling for *T*_1_.

*δ*: The simple correlation between *T*_1_ and *T*_2_.

*α*_1_: The reliability of *X*_1_ (var(*b*_1_*T*_1_)/var(*X*_1_)).

*α*_2_: The reliability of *X*_2_ (var(*b*_2_*T*_2_)/var(*X*_2_)).

(Note that in the special case where *X*_1_ and *X*_2_ measure the same true score—that is, *T*_1_ = *T*_2_ = *T*—then *δ* = 1 and *ρ*_1_ = *ρ*_2_ = *ρ*.)

The core incremental validity argument—i.e., the “argument for predictive utility”—claims that *T*_1_ is a useful predictor of *Y* even after controlling for *T*_2_. The corresponding null hypothesis for this argument, stated in terms of the statistical parameters just defined, is that *ρ*_1.2_ = 0. We reject this null hypothesis if we observe a significant partial correlation between *Y* and the measured variable *X*_1_, controlling for *X*_2_.

**Fig 3:**
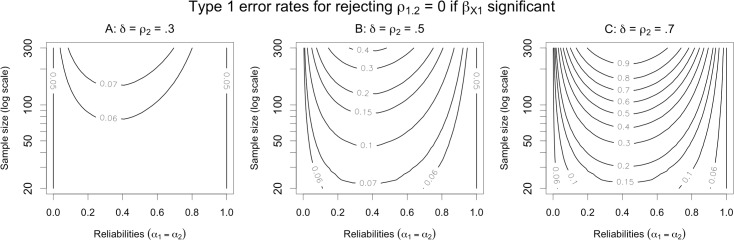
Contour plots of Type 1 error probabilities for the argument for predictive utility. The null hypothesis is that *T*_1_ has no partial relationship with *Y* after controlling for *T*_2_ (i.e., *ρ*_1.2_ = 0). The size of the true indirect effect of *T*_1_ on *Y* via *T*_2_ varies from small (panel A) to medium (panel B) to large (panel C).

Type 1 error rates for this argument are illustrated in Fig 3. The first thing to note is that if the control variable *X*_2_ is free of measurement error, the Type 1 error rate is, as expected, 5%. Although not illustrated in the plot, the error rate is also 5% if either *ρ*_2_ = 0 or *δ* = 0, either of which imply that the indirect effect of *T*_1_ on *Y* via *T*_2_ is 0, in which case there is no confounding influence to control for. However, if *X*_2_ is contaminated with any amount of measurement error, and there is any indirect effect of *T*_1_ on *Y* via *T*_2_, then the Type 1 error rate exceeds 5%. The extent by which the error rate exceeds 5% depends on three factors: the size of the indirect effect (*ρ*_2_*δ*), the sample size (*n*), and the reliability of *X*_2_ (*α*_2_).

The influence of the indirect effect size is straightforward: All else equal, as the indirect effect increases, the Type 1 error rate increases. When the indirect effect is small (both *ρ*_2_ and *δ* are modest; Fig 3A), the error rate is only slightly inflated. When the indirect effect is large (*ρ*_2_ and *δ* are large; Fig 3C), the error rate is very high for most representative values of *n* and *α*_2_. The intuitive explanation for this is that measurement unreliability makes it easier for the regression model to confuse the direct and indirect paths (i.e., to apportion variance in the outcome incorrectly between the various predictors). The larger the influence of the confounding covariate, the more variance can be misattributed to the predictor of interest, leading to an increase in Type I error.

The relationship between *n* and Type 1 error may be less obvious: all else equal, *as sample size increases*, *error rates also increase*. It is worth reflecting on this result, because it contravenes the received wisdom that larger samples mitigate most common statistical problems (e.g., as *n* grows, power to reject the null increases, parameter estimates become more precise, etc.). Indeed, we find that for studies involving thousands of participants and non-negligible indirect effects, rejection of the null hypothesis is a near certainty even when the null is in fact true (cf. Table 1). On reflection, the reason for this behavior becomes clear: as samples grow, power to detect *any* reliable association between the predictors and the outcome necessarily increases. This remains true even when measurement unreliability causes the model to confuse a common effect of two or more predictors with a unique effect of one predictor—as *n* grows, the model more confidently concludes that there is a reliable association between the predictor of interest and the outcome.

Finally, the effect of reliability on error rates is even less intuitive: there is a non-monotonic relationship, such that Type 1 error approaches 5% when reliability nears 0 or 1, but is highest when reliability is moderate. The error rate typically peaks when reliability is between 0.3 and 0.7, which is likely representative of many commonly used measures in the social sciences, particularly those that consist of a single item. However, even at a conventionally acceptable reliability of 0.7 or 0.8, the error rate can still be extremely high if the sample size and/or indirect effect are large. The non-monotonic effect of reliability has a compound explanation that becomes clear when one considers each extreme separately. When reliability is very low, the observed associations between all variables must be very small (i.e., power is very low), so the null cannot be rejected simply because it becomes almost impossible to detect any effect. Conversely, when reliability is very high, the model is able to avoid misattributing the effect of the covariate to the predictor of interest. In the middle, however, there exists a territory where effects are large enough to afford detection, but reliability is too low to prevent misattribution, leading to particularly high Type 1 error rates.

## Variations on the Same Theme

Thus far, we have focused analysis exclusively on the situation where one is attempting to show that a predictor of interest contributes incrementally to an outcome after controlling for a putative confound. As noted above, this situation is ubiquitous in the social sciences, and has been discussed extensively in previous work [8–10,22]. However, there are less obvious variants of the basic incremental validity argument that, despite being widely employed in some fields, have received less consideration. These more specialized arguments have the same basic structure laid out above: one infers relationships between latent constructs and outcomes of interest based on observed patterns of significant coefficients in a multiple regression of the outcome on composite scores thought to measure the latent constructs. These variants inherit all the statistical problems of the basic incremental validity argument, as well as additional problems related to the more complicated nature of the latent relationships being inferred.

One variant particularly common in the psychological sciences is what we might term the *argument for separable constructs*. Stated abstractly, it says: “If measurements of two constructs, *X* and *Z*, both significantly predict variation in an outcome *Y* while controlling for each other, then *X* and *Z* are distinct and independently useful constructs.” Real-world examples of this reasoning abound. In social psychology, theorists have argued for an important distinction between explicit attitudes and implicit attitudes [23–25] based partly on demonstrations that explicit and implicit attitude measures make statistically separable contributions to behavioral outcomes, notably including voting behavior [26–28]. In the individual differences literature, arguments for the existence of “multiple intelligences” often draw support from demonstrations that ability measures thought to tap different cognitive abilities make independent contributions to scholastic performance or other outcomes [29,30]. And in the neuroimaging literature, different brain regions or networks are often ascribed dissociable cognitive functions on the grounds that they each contribute unique variance to behavioral outcomes [31,32]. In all of these cases, it is tempting to conclude that the outcome in question is independently predicted by both of the predictors, which are thought to measure strongly related but conceptually distinct constructs. But a simpler interpretation that is often equally consistent with the data is that both predictors are simply noisy indicators of the same construct.

Our framework allows us to readily estimate Type 1 error rates for the argument for separable constructs. Here, the investigator seeks to establish the alternative hypothesis that both *ρ*_1.2_ ≠ 0 and *ρ*_2.1_ ≠ 0, which implies jointly rejecting the null hypotheses that *ρ*_1.2_ = 0 and *ρ*_2.1_ = 0. Formally, we reject the disjunctive null hypothesis “*ρ*_1.2_ = 0 or *ρ*_2.1_ = 0” if we observe that *both* multiple regression coefficients are significant.

**Fig 4:**
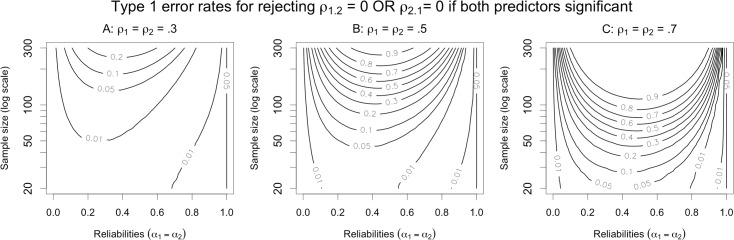
Contour plots of Type 1 error probabilities for the argument for separable constructs. The alternative hypothesis is that *both* of the predictors are separately related to the outcome, which implies the null hypothesis that either of the predictors is not related to the outcome. The magnitude of the true correlation between *Y* and *T* varies from small (panel A) to medium (panel B) to large (panel C).

Type 1 error rates for this argument are illustrated in Fig 4. As in the previous case, the error rate here approaches 5% as the two measured predictors approach perfect reliability. (The estimates of the regression coefficients are technically not identifiable when both predictors have reliability exactly equal to 1, since in that case the two predictors in the regression equation are perfectly collinear under the null hypothesis.) More realistically, when *X*_1_ and *X*_2_ are imperfectly reliable, the Type 1 error rate can vary dramatically depending on the sample size (*n*) and the true correlation between *Y* and *T* (*ρ*_1_ = *ρ*_2_ = *ρ*). In general, Type 1 error increases as *ρ* increases (compare panels in Fig 4)—with the exception that when reliability is high and sample size is low, increasing *ρ* can produce overly conservative error rates (e.g., less than .01). Similarly, increasing *n* generally increases Type 1 error. In fact, as the sample size approaches infinity, simultaneous rejection of both *β*_*X*1_ = 0 and *β*_*X*2_ = 0 becomes guaranteed even when the null hypothesis (*δ* = 1) is true, as long as *ρ* ≠ 0 and the reliabilities are between 0 and 1—both of which conditions are extremely probable in actual social science research. Furthermore, as Fig 4 makes apparent, the convergence of the error rate to 100% happens fairly quickly for many reasonable parameter values. For example, if the reliabilities are both .8 and we have *ρ* = .5 (so that each predictor’s simple, attenuated correlation with *Y* is about 0.45), then by the time the sample size reaches *n* = 300, the error rate has already surpassed 65%. We conclude that many previous findings in the social sciences are at high risk of having concluded that two constructs are distinct when they may not in fact be so.

Another variant of the incremental validity argument could be called the *argument for improved measurement* (or, less formally, the “mine is better” argument), which in its abstract form looks something like: “Our new measure of construct *X* is better than old measures of *X* because it is a better predictor of outcomes like *Y*, as evidenced by the fact that when we regress *Y* on both the old and new *X* measures, only the new *X* measure is significant.” For example, psychologists studying the relationship between feelings of shame and symptoms of depression have advanced a new measure of the construct of shame—the Experience of Shame Scale (ESS)—and argued for its utility in part by demonstrating that, when regressing a depressive symptom index on both ESS scores and scores on a previously used measure of shame—the Test of Self-Conscious Affect (TOSCA)—in a multiple regression model, the new ESS scores significantly predict the degree of depressive symptoms while the old TOSCA scores do not [33].

**Fig 5:**
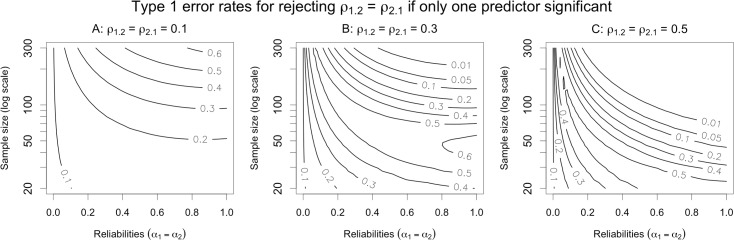
Contour plots of Type 1 error probabilities for the argument for improved measurement. The null hypothesis is that the two predictors have the same partial correlation with the outcome. The magnitude of the true partial correlation varies from small (panel A) to medium (panel B) to large (panel C). Varying *δ* does not have a very big impact on the error rates, so we fix it at *δ* = .5 in all three panels.

While this line of statistical reasoning has intuitive appeal, in most cases it actually provides very little evidence for the hypothesis that the new measure is more strongly related to the outcome than is the older measure. Again, we can quantify the false positive rate using our analytical framework. In this case, an investigator rejects the null hypothesis that *ρ*_1.2_ = *ρ*_2.1_, in favor of the alternative *ρ*_1.2_ ≠ *ρ*_2.1_, upon finding that one of the predictors in the observed multiple regression is significant while the other predictor is not. The Type 1 error surface for this argument, illustrated in Fig 5, is very unusual. The first thing to note is that, unlike with the previous two cases, here the error rate does not approach the nominal alpha level of 5% as the predictors approach perfect reliability. This is because the argument for improved measurement is actually based on flawed test logic: the correct procedure would directly test the difference *ρ*_1.2_ − *ρ*_2.1_ rather than separately testing the individual coefficients. Thus, while the unreliability of the predictors affects the error rates a lot (compare the panels of Fig 5), it is not really the main problem with the argument for improved measurement.

The second thing to note is that, unlike in the previous cases, here the error rate does not approach 100% as the sample size goes to infinity. Instead, the error rate increases until it reaches some maximum value that is typically in the 50%–65% range. After that point, the error rate then declines and begins to approach 0%, so that the test is *extremely* conservative (and hence underpowered) at large sample sizes. The intuition for this fact is that, when the sample size is very large, it is nearly certain that both of the predictors will be significant as long as there is some nonzero partial correlation, so observing only one predictor significant and the other nonsignificant would be extremely rare under the null hypothesis. The exact sample size at which the expected error rate reaches its maximum is a complicated, joint function of the reliabilities of the predictors and the strength of the partial correlations with the outcome. Two rules of thumb that can be gleaned from the plots are that the maximum error rate tends to be reached at a smaller sample size when (a) the reliabilities of the predictors are higher, and/or when (b) the partial correlations between the predictors and the outcome are stronger. One thing this analysis does make abundantly clear is that the Type 1 and 2 error rates associated with the argument for improved measurement are extremely poorly calibrated, so that the evidential value of rejecting this particular null hypothesis is often entirely unclear in practice.

Lastly, it is important to note while we have focused exclusively on Type 1 error probabilities within a frequentist hypothesis testing framework, essentially the same problem will arise no matter what statistical approach one uses (unless reliability is explicitly accounted for, as we discuss in the next section). For example, suppose one takes the view that the null hypothesis is almost never *exactly* true, and that researchers should instead focus on parameter estimation [34,35]. Consider the case of a true partial correlation that is very small but technically non-zero, *ρ*_1.2_ = 0.01. Given a large indirect effect size (*ρ*_2_ = *δ* = 0.7), modest reliabilities (*α*_1_ = *α*_2_ = 0.4), and a sample size of *n* = 100, the mean estimated incremental contribution is $r_{YX_{1}.X_{2}}=0.22$, with 95% of estimated values lying in [0.02, 0.40], which does not even include the true value *ρ*_1.2_. Thus, all of the conclusions we have drawn above generalize to a parameter estimation regime with little or no modification required. Nor is the problem restricted to frequentist approaches, as the same issues would arise for Bayesian models that fail to explicitly account for measurement error.

### Accounting for unreliability explicitly using structural models

The results presented above demonstrate that, in a wide range of very common research scenarios, incremental validity arguments based on multiple regression analysis run a very high risk of Type 1 error. Perhaps even more problematically, the error rates in question are typically unknown, because investigators often lack reasonable estimates of predictor reliability (e.g., econometric studies attempting to control for SES hardly ever estimate or report the reliability of the actual survey item(s) used to operationalize the SES construct). Of course, one could avoid these pitfalls by abstaining entirely from drawing construct-level claims on the basis of measurement-level models—and in general, we believe that researchers should always exercise extreme caution when ascribing latent-variable interpretations to observed variables. Ultimately, however, incremental validity arguments are statistical statements about the relationships between latent variables. As such, the most appropriate way to test such statements is to use latent variable approaches such as structural equation modeling (SEM), which can explicitly account for measurement unreliability.

In this section, we illustrate how an SEM approach can be used to support incremental validity claims through a reanalysis of a widely used personality data set—the Eugene-Springfield community sample [36,37], which involved a longitudinal study of nearly 1,000 adults who completed dozens of different self-report and behavioral measures over the course of 15 years. Here we use these data to test the incremental validity of two popular personality models in predicting respondents’ self-reported frequencies of a wide range of behaviors; however, the same general SEM approach can be used in virtually any situation where researchers wish to make construct-level incremental validity claims, and have some knowledge or estimate of the reliability of their predictors.

Our analysis focuses on the “Big Five” personality taxonomy—the most widely used personality model in modern psychology [38]. The Big Five model includes the traits of Openness to experience, Conscientiousness, Extraversion, Agreeableness, and Neuroticism. Proponents of the Big Five model have historically argued that these five personality factors explain most of the broad variation in people’s personalities, and that measurements of where people stand on these five factors can predict many major outcomes such as academic achievement, personality disorders, and work success [39–42]. More recently, however, some researchers have advanced a competing six-factor “HEXACO” model, which includes dimensions of Honesty-Humility, Emotionality, eXtraversion, Agreeableness, Conscientiousness, and Openness to experience [43]. Personality researchers have theorized a number of specific relationships between the six HEXACO factors and the Big Five factors [44]. Three of the HEXACO factors—Extraversion, Conscientiousness, and Openness to Experience—are thought to be essentially the same as the Big Five factors of the same name. The Emotionality and Agreeableness factors, on the other hand, are thought to be similar, but not identical, to the Big Five’s Neuroticism and Agreeableness factors. Finally, the HEXACO model introduces a sixth *Honesty-Humility* factor intended to capture a range of honesty- and modesty-related behaviors that are putatively overlooked by the Big Five [45].

This postulated pattern of construct-level relationships leads to three testable hypotheses about the relative contributions of Big Five and HEXACO traits to behavior. First, since the Extraversion, Conscientiousness, and Openness factors are thought to be conceptually identical in the NEO and HEXACO models, when we regress a behavioral outcome on both the NEO and HEXACO versions of these factors, we expect both regression coefficients to be nonsignificant. Second, since the Emotionality/Neuroticism and Agreeableness factors are thought to be conceptually different in the NEO and the HEXACO models, when we regress a behavioral outcome on both versions of these factors, we expect both the NEO and HEXACO predictors to have significant regression coefficients. Third, since the Honesty-Humility factor of the HEXACO is thought to explain additional variance in personality beyond the five NEO factors, participants’ Honesty-Humility scores should predict at least some behavioral outcomes even after controlling for their scores on the five NEO factors. The first two of these predictions correspond to what we called the “argument for separable constructs” (except that in the first case we predict that they are *not* separable) while the third prediction corresponds to the more common “argument for predictive utility.”

The standard way of testing these predictions is to compute sum or mean scores for each factor and then to enter these sum scores as simultaneous predictors in a multiple regression equation predicting the behavioral outcome of interest [46–48]. The SEM method of testing these predictions is similar, but rather than manually pre-computing sum scores, one instead specifies the measurement model for each factor as part of the structural regression model, so that the measurement error associated with each factor becomes an explicit part of the full model.

**Fig 6:**
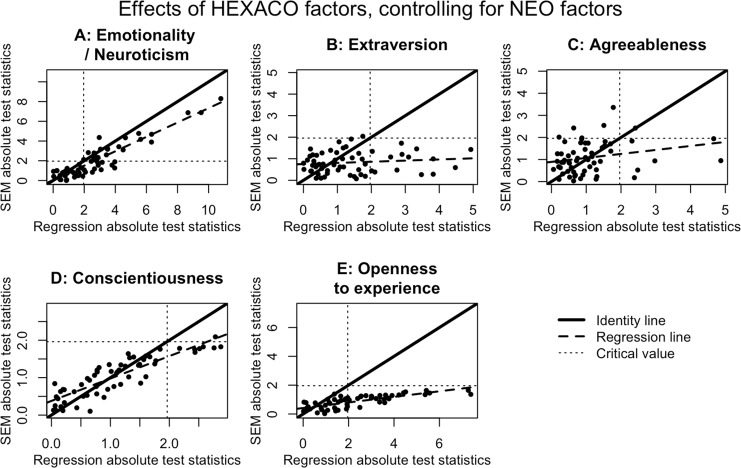
Test statistics from models regressing BRI outcomes on both the NEO and HEXACO versions of a factor. The test statistics are t-statistics for the regression models and z-statistics for the SEM models. BRI = Behavioral Report Inventory. SEM = Structural Equation Model.

**Fig 7:**
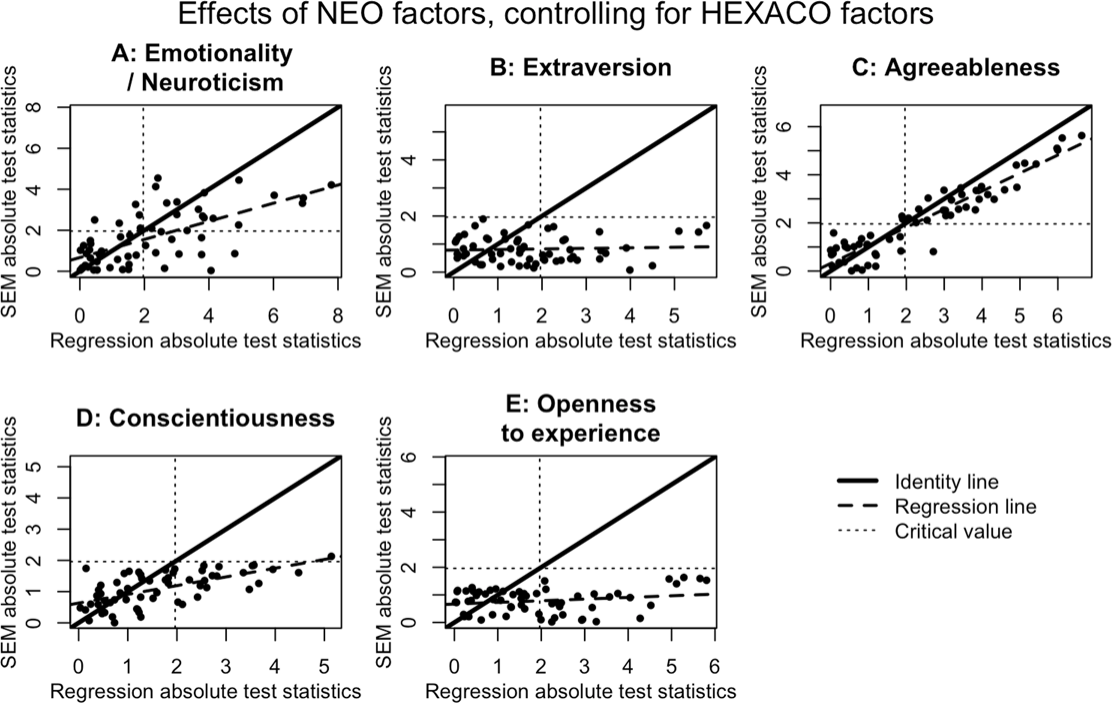
Test statistics from models regressing BRI outcomes on both the NEO and HEXACO versions of a factor. The test statistics are t-statistics for the regression models and z-statistics for the SEM models. BRI = Behavioral Report Inventory. SEM = Structural Equation Model.

We directly compared the conclusions produced by the two approaches using data from the publicly-available and widely-used Eugene-Springfield Community Sample [36,37]. Participants (n = 604) completed both the NEO-PI-R and the HEXACO-PI, as well as a “Behavioral Report Inventory” containing 400 descriptions of specific activities such as borrowing money, chewing gum, playing chess, or yelling at a stranger (for details, see [49]). Participants rated the frequency with which they engage in each activity, and Goldberg and colleagues aggregated the results into 60 distinct clusters. For each of these clusters, we conducted both regression-based and SEM analyses and compared the results. The results for the first and second predictions are illustrated for all 60 behaviors in Fig 6—which shows the absolute value of the test statistic (*t* for regression, *z* for SEM) for the HEXACO predictor, controlling for the NEO predictor—and in Fig 7—which shows the test statistic for the NEO predictor, controlling for the HEXACO predictor.

**Fig 8:**
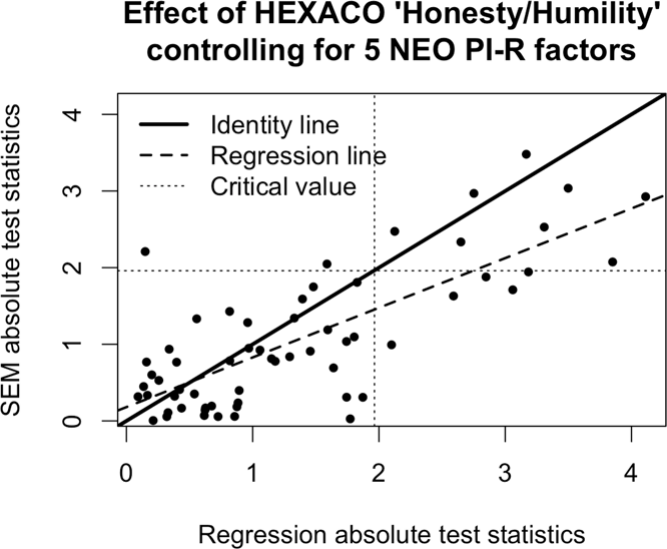
Test statistics from models predicting BRI outcomes. The test statistics are t-statistics for the regression models and z-statistics for the SEM models. BRI = Behavioral Report Inventory. SEM = Structural Equation Model.

For the multiple regression models, there are always many behaviors that appear to be independently predicted by both the HEXACO and NEO versions of all five factors. This should not be surprising given the results we reviewed in the last section demonstrating a very high Type 1 error rate for the multiple regression analyses; we expect to reject the null hypothesis a large proportion of the time whether it is true or false. By contrast, the SEM results produce more measured conclusions that support the theoretical predictions. The HEXACO and NEO versions of the Extraversion, Conscientiousness, and Openness to Experience factors almost never make separable contributions to any of the behaviors, consistent with our first prediction from above. For the Neuroticism/Emotionality factor, the SEM results look similar to the multiple regression results, with the HEXACO and NEO versions of the factor making separable contributions to predicting many of the behavioral outcomes. And for Agreeableness, the results are mixed: there are many behaviors in which the NEO factor predicts the outcome over and above the HEXACO factor, but only a few behaviors in which HEXACO significantly predicts the outcome over and above NEO. Thus, there is partial support of the second prediction from above. Finally, the results pertaining to the third prediction, which stated that Honesty-Humility should predict variation in behavior even after controlling for the five NEO factors, are shown in Fig 8. In this case the results from the SEM models look roughly similar to the results from the multiple regression models. In both cases there are a subset of the behaviors in which Honesty-Humility scores significantly predict variation over and above the five NEO factors. Thus, there is support for the third prediction from above as well.

### The Single Indicator Case

In all of our personality analyses above, we had multiple indicators of each construct (i.e., the individual questionnaire items). We were therefore able to empirically estimate the reliability with which each construct was measured, and the SEM models used these empirical reliability estimates to appropriately attenuate or disattenuate the parameter estimates for measurement error. However, in most cases, researchers do not have the luxury of multiple indicators of the constructs under study. For example, in a study of student outcomes in which we wish to control for the education level of students’ parents, we might have only a single survey item assessing parental education (e.g., “what is the highest level of education you have attained?”). Responses to such items typically contain a substantial amount of measurement error that, unfortunately, cannot be empirically estimated.

In such cases it is still possible to carry out SEM analyses like those presented above ([50] p. 168), [51], ([52] p. 276). The major difference is that, rather than relying on empirical estimates of reliability, we have to make assumptions about the level of reliability for any variables that we think contain measurement error. In this section we illustrate what such an analysis looks like, again using the Eugene-Springfield community sample. We test all of the same hypotheses as in the previous section, except that we pretend, for instructional purposes, that we have only a single indicator of each construct (the total scale score for each personality factor), and we examine how our results vary as a function of the assumptions we make about the reliability of the measures. For simplicity we focus on just one of the 60 BRI clusters—the frequency of self-reported illegal drug use.

**Fig 9:**
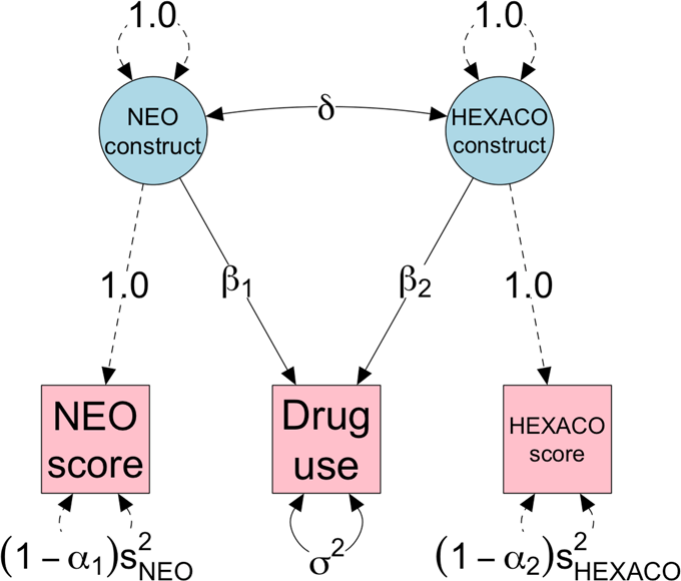
Path diagram for a SEM predicting drug use, allowing for specified degrees of reliability in the observed NEO and HEXACO scores. Circle nodes represent latent variables, square nodes represent observed variables, solid lines represent paths or variances to be estimated from the data, and dashed lines represent paths or variances that are fixed to constant, a priori values. SEM = Structural Equation Model.

Fig 9 shows a path diagram representing the appropriate structural equation model to fit to these data. We define two correlated latent variables that are indicated by the observed NEO and HEXACO sum scores, and then regress the drug use outcome simultaneously on these latent predictors. Of course, the reliability of the NEO and HEXACO scores cannot be estimated from the data since we are considering them to each consist of only a single measurement—in terms of the model, the variances of the residuals perturbing the observed predictors are not identifiable. Therefore, to model the effects of unreliability of the predictors, we fix the loadings of both indicators to 1.0 and fix the variances of their residuals to $\left(1-\alpha_{j}\right)s_{j}^{2}$, where *α*_*j*_ is the assumed reliability of the *j*th predictor and $s_{j}^{2}$ is the sample variance of that predictor. For example, the observed variance of the NEO neuroticism scores is 0.42, so to constrain the reliability of the neuroticism scores to be, say, 0.4, we would constrain its residual variance to be (1−.4)0.42 = 0.25.

**Fig 10:**
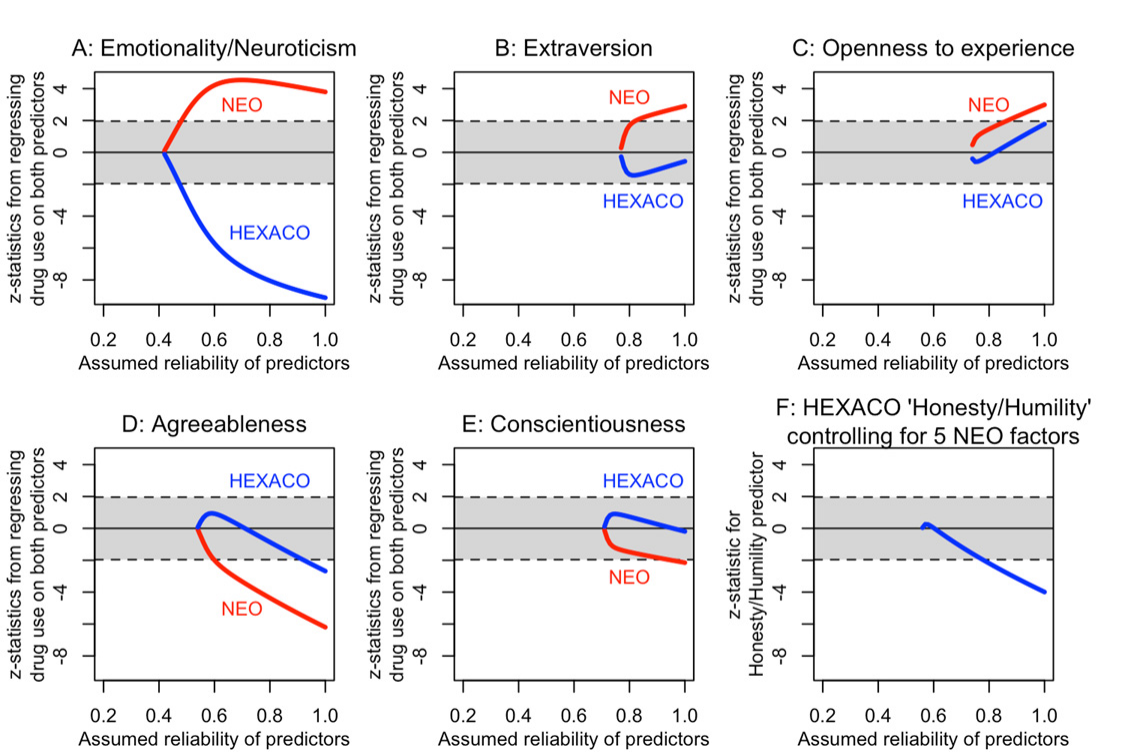
Test statistics as a function of assumed reliability. The shaded region gives the range within which the test statistics are nonsignificant. In each model, assuming reliabilities below a certain value invariably caused the model to fail to converge or to yield an inadmissible solution (i.e., impossible correlation matrices for the latent variables); we only plot the results for reliability values that successfully converge on stable estimates.

The results obtained from this procedure depend on the values of *α*_*j*_ that we have assumed. Our recommendation is therefore to run the procedure for a range of assumed values of *α*_*j*_ and investigate how the statistical conclusion varies as a function of the assumed reliability. This idea is illustrated in Fig 10, where we repeat all the analyses from Figs 6, 7 and 8, but vary the assumed reliability of the HEXACO and NEO sum score predictors from 0.2 to 1 (see Fig caption for additional details). The values plotted on the curves in panels A through E are the z-statistics from the structural equation model output for testing the null hypotheses that *β*_1_ = 0 and *β*_2_ = 0, that is, for testing the partial effect of HEXACO scores on drug use, controlling for the NEO scores, and vice versa (analogous to Figs 6 and 7). The z-statistics plotted in panel F are from a structural equation model with 6 predictors, testing the null hypothesis that HEXACO honesty/humility scores significantly predict drug use over and above the five NEO scores (analogous to Fig 8).

The results of this analysis show that our conclusions about whether the HEXACO and NEO factors are incrementally valid predictors of drug use sometimes depend on our assumptions about the reliability of the sum scores, and sometimes do not. For example, the NEO Neuroticism and HEXACO Emotionality predictors both significantly predict drug use as long as we assume their reliabilities are at least 0.5 (a reasonable assumption given prior literature). By contrast, for the Extraversion, Openness to Experience, and Conscientiousness factors, there is no level of reliability for which both of the predictors significantly predict drug use controlling for one another. For Agreeableness, both the NEO and HEXACO predictors are significant only if we assume that their reliabilities are at least *α* ≥ .92, which is not impossible, but seems highly unlikely a priori. And in fact, since in this example we actually do have multiple indicators, if we estimate the reliabilities empirically we get estimates of .73 for NEO agreeableness and .79 for HEXACO agreeableness—so the full multi-indicator SEM analysis would not have found evidence that NEO and HEXACO agreeableness are incrementally valid predictors of drug use. Finally, in panel F we see that the HEXACO Honesty-Humility scores only significant predict drug use over and above NEO scores if the reliabilities are assumed to be at least *α* ≥ .78, which could plausibly be the case, although it is far from certain. Thus, the results of this analysis are somewhat ambiguous. Once again, if we estimate the reliabilities empirically we get estimates that range from .66 to .79 with an average of $\bar{\alpha }=.74$. So the full SEM model would have yielded at best weak evidence for incremental validity as well.

## Statistical Power of Incremental Validity Arguments Using SEM

The reanalyses presented above make it clear that, when arguing for incremental validity, the reliability of the predictors matters. Seemingly strong evidence for incremental validity based on a multiple regression model that ignores measurement error can easily disintegrate when one uses more appropriate, SEM-based methods that account for measurement error in the predictors. This observation naturally raises an important question: what kind of statistical power do incremental validity arguments have when they are based (correctly) on SEM rather than multiple regression?

**Fig 11:**
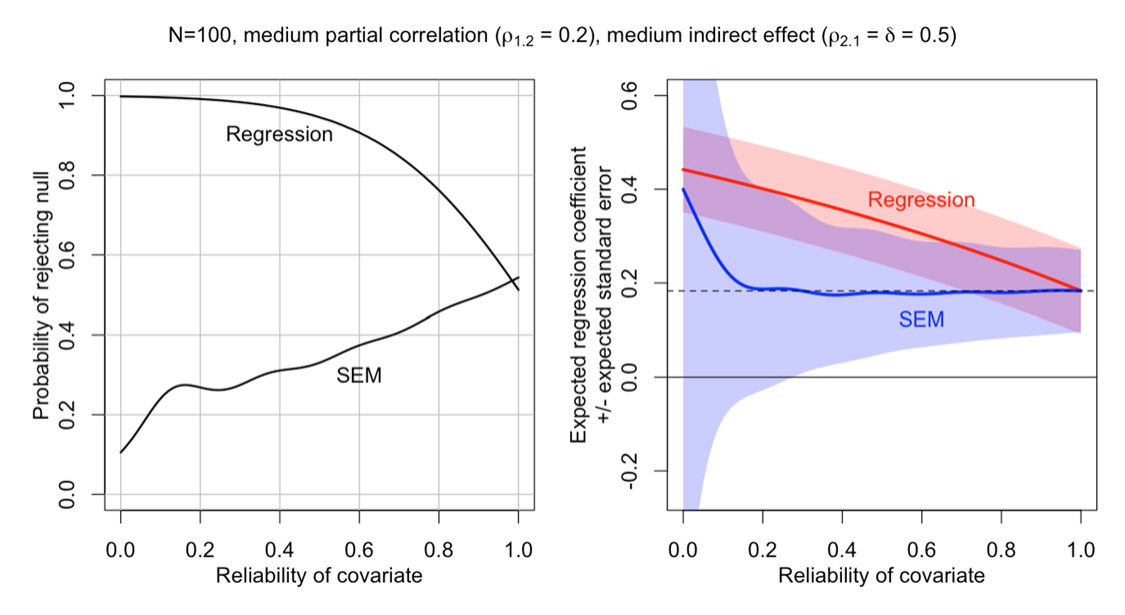
Incremental validity in multiple regression vs. SEM. The SEM results are from a simulation using 300,000 iterations. The multiple regression results are computed analytically. The SEM line in the left panel is a smoothed curve derived from fitting a generalized additive model with a binomial response to the simulation results tracking whether the null hypothesis was rejected. In the right panel, the SEM line and shaded region are based on first applying rolling medians of width 101 to the simulated regression coefficients and standard errors (to reduce the distorting influence of extreme outlying parameter estimates occurring particularly at low reliability values), and then fitting a generalized additive model to these rolling medians. SEM = Structural Equation Model.

Naively, one might suppose that an SEM analysis should be only modestly more conservative than its multiple regression equivalent. However, multiple regression and SEM respond very differently to the presence of measurement error in a covariate. As illustrated in Fig 11, adding an increasingly unreliable covariate to an SEM model causes the standard error of the parameter estimate for the predictor of interest to grow larger and larger. The intuition for this behavior is that the model must adjust the parameter estimate to account for the overlap with the covariate, but as the covariate’s unreliability increases, it becomes increasingly unclear exactly how much of an adjustment is required. This increasing uncertainty is reflected in the increasing standard error. By contrast, multiple regression will typically show the opposite trend: the more unreliable the covariate, the more the multiple regression actually capitalizes on this unreliability by conflating the direct and indirect effects of the predictor of interest, leading to biased, inconsistent parameter estimates and inflated test statistics [6]. The net effect is that, as the reliability of a covariate falls, it typically becomes easier to reject the null with multiple regression (resulting, as we have already seen, in very high false positive rates when the null is true), but harder to reject the null with SEM. The latter is the correct behavior, as it reflects our expectation that introducing additional measurement error into a set of regression equations should increase the uncertainty in parameter estimates and correspondingly attenuate the test statistics for hypothesis tests on those parameters. The upshot is that shifting from multiple regression to SEM should increase the sample size required to support incremental validity claims. The key question is by how much.

To find out, we conducted a simulation. We generated random data according to a structural equation model identical in structure to the model shown in Fig 9. The reliability of the focal predictor of interest was always kept at 1.0, while the assumed reliability of the covariate was set to either perfect (*α* = 1; equivalent to multiple regression), high (*α* = .8; a typical reliability for an aggregate of multiple items), or low (*α* = .4; a typical reliability for a single item). (Adding measurement error to the focal predictor would, of course, simply diminish the statistical power even further and lead the required sample sizes to be even larger.) We assumed a relatively large indirect effect of the focal predictor via the covariate, with *δ* = *ρ*_2.1_ = .7. We reasoned that, in the real word, the situations where it occurs to the researcher that it might be important to control for a particular covariate are precisely those in which the covariate has a large indirect effect, so that large indirect effects are probably common in much actual research. We varied the size of the partial correlation between the focal predictor and the outcome between *ρ*_1.2_ = 0 (to verify that the SEM can keep the Type 1 error rate at approximately the nominal alpha level of 5%) to *ρ*_1.2_ = .3, in increments of 0.1. For each parameter combination we ran the simulation 30,000 times, each time drawing sample sizes from a distribution uniform on the log scale from *n* = 50 to *n* = 5000.

**Fig 12:**
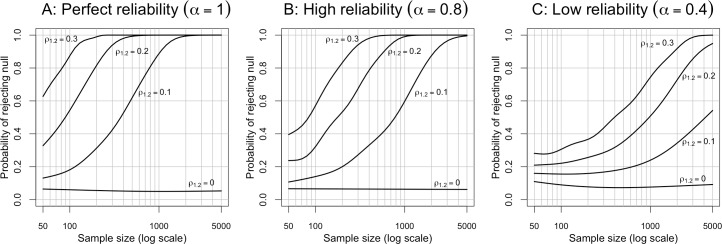
Power to detect incremental validity using SEM. The lines in each panel are smoothed curves derived from fitting generalized additive models with a binomial response to the simulation results. SEM = Structural Equation Model.

The results of the simulation are shown in Fig 12. Panel A, in which the covariate is perfectly reliable, shows a relatively happy situation: with effect sizes of *ρ*_1.2_ = .3, .2, or .1, achieving 80% power requires sample sizes of *n* ≈ 80, 200, or 800, respectively. These are equivalent to the power results for multiple regression, and we suspect that most researchers’ intuitions about statistical power are calibrated to a situation similar to this one. We also see that the Type 1 error rate is maintained at 5%. In panel B, where we now introduce just a relatively small amount of measurement error to the covariate, we can see that the required sample sizes increase substantially: with effect sizes of *ρ*_1.2_ = .3, .2, or .1, achieving 80% power now requires sample sizes of *n* ≈ 180, 400, or 1600, respectively. The Type 1 error rates are still maintained at 5%. Finally, in panel C, where the covariate is measured with a substantial amount of error—as is likely typical with single indicator covariates widely used across many fields—the required sample sizes are now very large. With effect sizes of *ρ*_1.2_ = .3 or .2, achieving 80% power requires sample sizes of *n* ≈ 1200 or 2300, respectively. The required sample size for *ρ*_1.2_ = .1 is too large to even show within the plot margins, but it seems to be well into the tens of thousands. We also see that, in the low reliability case, there is some slight elevation of the Type 1 error rate, although it does not appear to go much beyond 10%.

For comparison, we also conducted a simulation involving a small indirect effect size of *δ* = *ρ*_2.1_ = .3. In this simulation, the required sample sizes to achieve 80% power with direct effect sizes of *ρ*_1.2_ = .3, .2, or .1 were about *n* ≈ 80, 200, or 800, respectively, for both perfect reliability and high reliability. For low reliability, the required sample sizes were about *n* ≈ 110, 300, or 1000, respectively. We note that such estimates are probably much too optimistic for most real-world situations, as it is rare for a single predictor to exert nearly all of its influence on the outcome via the direct path, and independently of other possible covariates.

## Discussion

To most social scientists, observed variables are essentially just stand-ins for theoretical constructs of interest. The former are only useful to the extent that they accurately measure the latter. Accordingly, it may seem natural to assume that any statistical inferences one can draw at the observed variable level automatically generalize to the latent construct level as well. The present results demonstrate that, for a very common class of incremental validity arguments, such a strategy runs a high risk of failure. The scope of the problem is considerable: literally hundreds of thousands of studies spanning numerous fields of science have historically relied on measurement-level incremental validity arguments to support strong conclusions about the relationships between theoretical constructs. The present findings inform and contribute to this literature—and to the general practice of “controlling for” potential confounds using multiple regression—in a number of ways.

First, we show that the traditional approach of using multiple regression to support incremental validity claims is associated with extremely high false positive rates under realistic parameter regimes. Researchers relying on such arguments will thus often conclude that one construct contributes incrementally to an outcome, or that two constructs are theoretically distinct, even when no such conclusion is warranted. Of course, this general problem is not novel, and has been discussed in a number of literatures [8–10]—most extensively, under the heading of “residual confounding” in epidemiology [3,5]. However, previous treatments have typically focused on circumscribed aspects of the problem or applications to specific domains. Here we have introduced a general formal framework that can be easily used to derive expected false positive rates for any combination of reliabilities and effect sizes, expressed in terms of either simple or partial correlations. As a complement, we also provide a web application that enables researchers to obtain these quantities using a simple point-and-click interface (http://jakewestfall.org/ivy/). Application of our framework to a wide range of realistic scenarios demonstrates that key parameters interact with one another in complex, and sometimes counterintuitive, ways. For example, we find that false positive rates typically increase with sample size, and typically peak when reliability is moderate rather than when it is very low or very high. In general, we find that the probability of spurious inference approaches 100% much more quickly than one might imagine, and under realistic parameter regimes will typically be several times the nominal rate of 5%.

Second, we demonstrate that the problem has a principled solution: inferences about the validity of latent constructs should be supported by latent-variable statistical approaches that can explicitly model measurement unreliability. Researchers in a position to measure constructs using multiple indicators can rely on well-established structural equation modeling techniques to support construct-level inferences; however, we also show how even when only a single indicator is available, researchers can use an SEM approach to estimate what level of reliability must be assumed in order to support the validity of one’s inferences ([50] p. 168), [51], ([52] p. 276)—providing important insights into the plausibility and/or boundary conditions of posited relationships. A major strength of the latter approach is that it can be readily applied to existing datasets, thus enabling researchers to re-evaluate previous incremental validity claims with measurement unreliability taken into account.

Lastly, we address an important question that, to our knowledge, has not been previously investigated in the literature: what kind of sample sizes are required to achieve adequate statistical power to detect incremental contributions at the latent variable level? While the answer will necessarily vary across contexts, we show that, under realistic conditions likely to apply fairly widely, statistical power to establish incremental validity at the construct level is often shockingly low. In particular, when the unique contribution of the construct of interest is relatively small, a study can easily require tens of thousands of participants to establish that construct’s incremental validity. Even when the effect is moderate to large, achieving adequate power in the presence of moderately unreliable covariates will often require hundreds of participants. Moreover, our analyses focused only on the case where a single covariate is included in the model. The inclusion of additional imperfectly measured covariates—as is common in real-world analyses—will generally make detection of incremental validity even more difficult.

## Conclusion

Taken as a whole, our results demonstrate that drawing construct-level inferences about incremental validity is considerably more difficult than most researchers recognize. We do not think it is alarmist to suggest that many, and perhaps most, incremental validity claims put forward in the social sciences to date have not been adequately supported by empirical evidence, and run a high risk of spuriousness. By this we do not mean to suggest that such claims are *wrong*, but simply that the modal analytical strategy of controlling for one or more covariates in a multiple regression cannot provide adequate evidence for a construct-level incremental validity claim under realistic conditions where variables are measured unreliably. Our hope is that greater appreciation of the inferential dangers of confusing measures with constructs [21] will lead researchers to adopt statistical approaches like SEM that provide appropriately calibrated evidence for incremental validity claims.

## Supporting Information

S1 Appendix Derivation of statistical properties of incremental validity. We derive the probabilities of rejecting different combinations of regression coefficients as a function of (1) the simple or partial correlations among the outcome and the latent predictors, (2) the reliabilities, and (3) the
(DOCX)
