# Supplementary material for: Statistically Controlling for Confounding Constructs Is Harder than You Think
Source: PLoS One. 2016 Mar 31;11(3):e0152719. doi: 10.1371/journal.pone.0152719 (PMC4816570; doi:10.1371/journal.pone.0152719)
Supplement: S1 Appendix — We derive the probabilities of rejecting different combinations of regression coefficients as a function of (1) the simple or partial correlations among the outcome and the latent predictors, (2) the reliabilities, and (3) the (DOCX) [file pone.0152719.s001.docx]

***Statistically controlling for confounding constructs is harder than you think***

Jacob Westfall and Tal Yarkoni

**SUPPLEMENTAL APPENDIX S1**

**Derivation of statistical properties of incremental validity**

Consider a regression of an outcome *Y* on two true scores $T_{j}$,

$$Y={\beta_{T0}+\beta_{T1}T}_{1}+{\beta_{T2}T}_{2}+ e_{T},$$

with $e_{T}$ a random disturbance term (the subscripts indexing people are omitted for simplicity). Rather than observing the predictors $T_{j}$ directly, we instead observe two imperfectly measured indicators $X_{j}=b_{j}T_{j}+ e_{j}$, so that the regression we actually observe is

$$Y=\beta_{X0}+{\beta_{X1}X}_{1}+\beta_{X2}X_{2}+ e_{X}.$$

From these regressions we define the following parameters:

$\rho_{1}$: The simple correlation between *Y* and $T_{1}$.

$\rho_{2}$: The simple correlation between *Y* and $T_{2}$.

$\delta$: The simple correlation between $T_{1}$ and $T_{2}$.

$\alpha_{1}$: The reliability of $X_{1}$ $\left( \text{var}\left( {b_{1}T}_{1} \right)/\text{var}\left( X_{1} \right) \right)$.

$\alpha_{2}$: The reliability of $X_{2}$ $\left( \text{var}\left( {b_{2}T}_{2} \right)/\text{var}\left( X_{2} \right) \right)$.

*n*: The total sample size.

Note that there is the possibility that $T_{1}=T_{2}=T$, in which case $\delta=1$ and $\rho_{1}=\rho_{2}=\rho$. For simplicity, we assume that all observed variables are standardized to unit variance. Then we can write the full correlation matrix of all variables as follows:

|  | $Y$ | $T_{1}$ | $T_{2}$ | $X_{1}$ | $X_{2}$ |
| --- | --- | --- | --- | --- | --- |
| $Y$ | $1$ | $\rho_{1}$ | $\rho_{2}$ | $\rho_{1}\sqrt{\alpha_{1}}$ | $\rho_{2}\sqrt{\alpha_{2}}$ |
| $T_{1}$ | $\rho_{1}$ | $1$ | $\delta$ | $\sqrt{\alpha_{1}}$ | $\delta\sqrt{\alpha_{2}}$ |
| $T_{2}$ | $\rho_{2}$ | $\delta$ | $1$ | $\delta\sqrt{\alpha_{1}}$ | $\sqrt{\alpha_{2}}$ |
| $X_{1}$ | $\rho_{1}\sqrt{\alpha_{1}}$ | $\sqrt{\alpha_{1}}$ | $\delta\sqrt{\alpha_{1}}$ | $1$ | $\delta\sqrt{\alpha_{1}\alpha_{2}}$ |
| $X_{2}$ | $\rho_{2}\sqrt{\alpha_{2}}$ | $\delta\sqrt{\alpha_{2}}$ | $\sqrt{\alpha_{2}}$ | $\delta\sqrt{\alpha_{1}\alpha_{2}}$ | $1$ |

Our goal is to find, given the parameters above, (1) the probability of simultaneously rejecting the null hypothesis that $\beta_{X1}=0$ and rejecting the null hypothesis that $\beta_{X2}=0$, and (2) the probability of simultaneously rejecting the null hypothesis that $\beta_{X1}=0$ and *failing* to reject the null hypothesis that $\beta_{X2}=0$. (Also of interest is the probability of rejecting $\beta_{X1}=0$ *regardless* of whether $\beta_{X2}=0$ is rejected, but note that this is simply the sum of the two probabilities just mentioned.) To find these two probabilities, we will consider the joint distribution of the *t*-statistics associated with $\beta_{X1}$ and $\beta_{X2}$ in the observed regression. The cumulative distribution function of the bivariate noncentral *t*-distribution takes as arguments a 2×1 vector of noncentrality parameters $\boldsymbol{\mu}$, a 2×2 matrix of scale parameters $\boldsymbol{\Sigma}$, and the degrees of freedom $\nu$. So finding the two probabilities above entails expressing all the elements of $\boldsymbol{\mu}$ and $\boldsymbol{\Sigma}$ in terms of simple correlations from the table above.

The noncentrality parameters in $\boldsymbol{\mu}$ can be written in terms of the two partial correlation coefficients $r_{YX_{j}.X_{k}}$, denoting the correlation between *Y* and $X_{j}$ controlling for $X_{k}$, as follows:

$$\boldsymbol{\mu}=\left[ \begin{aligned} \sqrt{\frac{\nu r_{YX_{1}.X_{2}}^{2}}{1- r_{YX_{1}.X_{2}}^{2}}} \\ \sqrt{\frac{\nu r_{YX_{2}.X_{1}}^{2}}{1- r_{YX_{2}.X_{1}}^{2}}} \end{aligned} \right].$$

The partial correlation between *Y* and $X_{j}$, controlling for $X_{k}$, can in turn be written in terms of the three simple correlations among the variables as

$$r_{YX_{j}.X_{k}}= \frac{r_{YX_{j}}- r_{YX_{k}}r_{X_{j}X_{k}}}{\sqrt{1-r_{YX_{k}}^{2}}\sqrt{1-r_{X_{j}X_{k}}^{2}}}.$$

where $r_{YX_{j}}$ refers to the simple correlation between *Y* and $X_{j}$, and so on. The only other term in $\boldsymbol{\mu}$ is the degrees of freedom for the multiple regression,$\nu=n-3$.

The matrix of scale parameters $\boldsymbol{\Sigma}$ corresponds to the variance-covariance matrix of the regression coefficients associated with $X_{1}$ and $X_{2}$ in the observed regression of *Y* on the two indicators. So the diagonal elements are $\text{var}\left( \hat{\beta}_{X1} \right)$ and $\text{var}\left( \hat{\beta}_{X2} \right)$, and the off-diagonal element is $\text{cov}\left( \hat{\beta}_{X1},\hat{\beta}_{X2} \right)$. The full matrix can be written as

$$\boldsymbol{\Sigma}= \left[ \begin{matrix} \frac{\text{var}\left( e_{X} \right)}{\nu\left( 1- r_{X_{1}X_{2}}^{2} \right)\text{var}\left( X_{1} \right)} & \frac{\text{var}\left( e_{X} \right)\text{cov}\left( X_{1}, X_{2} \right)}{n\left( {\text{cov}\left( X_{1}, X_{2} \right)}^{2}-\text{var}\left( X_{1} \right)\text{var}\left( X_{2} \right) \right)} \\ \frac{\text{var}\left( e_{X} \right)\text{cov}\left( X_{1}, X_{2} \right)}{n\left( {\text{cov}\left( X_{1}, X_{2} \right)}^{2}-\text{var}\left( X_{1} \right)\text{var}\left( X_{2} \right) \right)} & \frac{\text{var}\left( e_{X} \right)}{\nu\left( 1- r_{X_{1}X_{2}}^{2} \right)\text{var}\left( X_{2} \right)} \end{matrix} \right].$$

Recall that the variances of $X_{1}$ and $X_{2}$ are 1 by assumption. The only other term in $\boldsymbol{\Sigma}$ that we have not already seen is $\text{var}\left( e_{X} \right)$, the variance of the residuals from the observed multiple regression of *Y* on $X_{1}$ and $X_{2}$. To find this we first write

$$\text{var}\left( e_{X} \right)=\left( 1-R_{X}^{2} \right)\text{var}\left( Y \right).$$

The variance of *Y* is 1 by assumption, and the term $R_{X}^{2}$ is the coefficient of determination from the observed multiple regression, which can be written in terms of the simple correlations as

$$R_{X}^{2}=\frac{r_{YX_{1}}^{2}+r_{YX_{2}}^{2}-2r_{{YX}_{1}}r_{YX_{2}}r_{X_{1}X_{2}}}{1-r_{X_{1}X_{2}}^{2}}.$$

We are now in a position to compute the two probabilities described at the beginning of this Appendix, given the three simple correlations among *Y*, $X_{1}$ and $X_{2}$. One final step is to show how one can compute these probabilities given *partial* correlations among *Y*, $T_{1}$ and $T_{2}$, which we do in the main text in various places. There are essentially three cases to be considered: (1) the case where we are given two simple correlations and one partial correlation; (2) the case where we are given three partial correlations; and (3) the case where we are given two partial correlations and one simple correlation.

For case (1), we can compute the one missing simple correlation by solving the partial correlation formula given previously for the corresponding simple correlation, which yields

$$r_{AB}=r_{AB.C}\sqrt{1-r_{AC}^{2}}\sqrt{1-r_{BC}^{2}}+r_{AC}r_{BC}.$$

After solving for the $r_{AB}$ term, we now have three simple correlations and can proceed using all the methods described above. For case (2), we can compute each of the three simple correlations $r_{AB}$ from the three partial correlations $r_{AB.C}$, $r_{AC.B}$ and $r_{BC.A}$ using

$$r_{AB}= \frac{r_{AB.C}+ r_{AC.B}r_{BC.A}}{\sqrt{\left( r_{AC.B}^{2}-1 \right)\left( r_{BC.A}^{2}-1 \right)}}.$$

Again, after applying this for each of the three simple correlations, we can use the methods described previously. Finally, for case (3), we first compute the one missing partial correlation by solving the equation just given for the $r_{AB.C}$ term, which yields

$$r_{AB.C}=r_{AB}\sqrt{\left( r_{AC.B}^{2}-1 \right)\left( r_{BC.A}^{2}-1 \right)}-r_{AC.B}r_{BC.A}.$$

After solving for $r_{AB.C}$, case (3) is reduced to case (2), which we can handle as described above.
